# Genetic Polymorphisms in the RAD51 Gene with a Risk of Head and Neck Cancer and Esophageal Cancer: A Meta-Analysis

**DOI:** 10.1155/2019/2789035

**Published:** 2019-12-05

**Authors:** Lin Li, Xue Zhang, Zhong-Ti Zhang

**Affiliations:** ^1^VIP Department, School of Stomatology, China Medical University, Shenyang, Liaoning 110002, China; ^2^VIP Department, School of Stomatology, China Medical University, 117 North Nanjing Street, Heping District, Shenyang, Liaoning 110002, China

## Abstract

**Background:**

The role of RAD51 gene polymorphisms with the development of head and neck cancer (HNC) and esophageal cancer (EC) remains controversial. This meta-analysis was conducted to evaluate the correlation between the RAD51 polymorphisms and these two cancers quantitatively.

**Methods:**

Databases of PubMed, Web of Science, and Embase were used to search relevant papers prior to August 17, 2019. STATA 11.0 was performed to observe the correlation.

**Results:**

Ten relevant papers were enrolled in our analysis. Overall, a significant correlation was observed between the rs1801320 polymorphism and the increased risk of these two cancers (OR = 1.32, 95%CI = 1.03‐1.71 for C vs. G; OR = 1.50, 95%CI = 1.03‐2.19 for CG vs. GG; and OR = 1.44, 95%CI = 1.05‐1.99 for CC+CG vs. GG). In subgroup analyses, an increased risk was found for EC (OR = 2.07, 95%CI = 1.01‐4.25 for C vs. G; OR = 2.08, 95%CI = 1.17‐3.71 for CC vs. GG; and OR = 1.78, 95%CI = 1.00‐3.15 for CC vs. CG+GG), but not for HNC. Moreover, our analysis revealed that no statistical evidence of correlation was discovered between the polymorphism of rs1801321 and the increased risk of HNC. However, stratified analysis based on ethnicity suggested that rs1801321 polymorphism was related to the decreased risk of HNC among Caucasians (OR = 0.82, 95%CI = 0.72‐0.95 for T vs. G).

**Conclusions:**

rs1801320 polymorphism was strongly associated with the risk of these two associated cancers, especially with esophageal cancer. Moreover, our results revealed that rs1801321 polymorphism was correlated to the decreased risk of HNC among Caucasians.

## 1. Introduction

Head and neck cancer (HNC) is the sixth most common cancer [1], which includes oral cancer, nasopharyngeal carcinoma, pharyngeal cancer and laryngeal neoplasm. Most of HNC patients are over 50-60 years old, but the number of younger patients has increased recently [2]. According to statistics, an estimated 650,000 new cases of HNC and 350,000 deaths occur each year worldwide [3]. Esophageal cancer (EC) is one of the most common carcinomas that ranks eighth in incidence rate and ranks sixth in mortality rate [4]. In recent decades, many studies have reported that patients with HNC were at a higher risk of developing concomitant EC than general persons, particularly cancers of the oral cavity [5–12]. Besides, a few papers put forward that patients with EC also have an increased risk of a second OC [13, 14]. The bidirectional association between OC and EC was well established by Chuang et al. [14] and Lee et al. [15], no matter which one occurs first.

The strong correlation between the incidence of HNC and the incidence of EC might be because these two cancers share some same factors. Although the primary risk factors for HNC and EC are smoking and heavy alcohol intake [16, 17], only a small minority of these subjects will develop HNC or EC [18–20], suggesting that genetic susceptibility might also be strongly associated with the development of these two cancers [20–25].

RAD51 gene, mapped to chromosome 15q15.1 in people, belongs to the system of DNA repair gene [26]. RAD51 protein encoded by the gene has a significant effect on the repairment of damaged DNA and maintaining genomic integrity [27]. 135 G/C (rs1801320) and 172 G/T (rs1801321) are two common RAD51 single nucleotide polymorphisms (SNPs), which might influence mRNA stability and relate to altered translational efficiency [28]; thus, these two gene polymorphisms might lead to carcinogenesis.

To date, a variety of articles have been conducted to assess the correlation between the RAD51 gene polymorphisms and the risk of HNC and EC [29–39]. However, the results are still inconsistent. For instance, Sun et al. [39] demonstrated G135C polymorphism in RAD51 gene was strongly related to EC, while Zhang et al. [37] did not discover the significant relationship between EC and polymorphisms in RAD51. So the meta-analysis was conducted to examine the correlation between the polymorphisms in RAD51 gene and the susceptibility to HNC and EC.

## 2. Material and Methods

### 2.1. Selection of Relevant Papers

Relevant papers were searched in databases of PubMed, Web of Science, and Embase prior to August 17, 2019. Selection strategy was carried out by combination of the following terms: “RAD51”, “135G/C”, “rs1801320”, “172G/T”, “rs1801321”, “polymorphism”, “polymorphisms”, “variant”, “mutation”, “SNP”, “HNC”, “head and neck”, “oral”, “oral cavity”, “pharyngeal”, “laryngeal”, “nasopharyngeal”, “oropharyngeal”, “laryngopharyngeal”, “hypopharyngeal”, “esophageal”, “oesophageal”, “cancer”, “carcinoma”, “tumor”, “tumour”, “malignancy”, and “neoplasm”. Furthermore, references cited by all the retrieved papers were checked to identify potentially relevant articles.

### 2.2. Inclusion and Exclusion Criteria

Our meta-analysis was performed follow the recommendations of the Preferred Reporting Items for Systematic Reviews and Meta-Analyses statement [40].

Papers which met all the following criteria were enrolled in the present meta-analysis: (1) associated with the correlation between the RAD51 SNP and the EC or HNC, (2) case-control studies, and (3) sufficiency of the data provided by the paper for estimating the correlation. Publications satisfying one of the following criteria were excluded: (1) unavailability of full text, (2) no sufficient information on data reported, and (3) duplicated papers, reviews, editorials, case reports, commentaries, and case-only studies.

### 2.3. Data Extraction and Quality Assessment

The following information on data were fetched by two writers, respectively, from each enrolled study: name of primary author, publication year, country where paper was performed, ethnicity of the study subjects, counts of case groups and control groups, source of the control groups, genotyping methods, genotype and allele for case and control frequencies, type of tumor, and *P* value of the Hardy-Weinberg equilibrium (HWE) in controls. Cases of disagreement were resolved by two writers. Quality assessment of each paper enrolled in the analysis was performed by the Newcastle-Ottawa Scale (NOS) criteria [41].

### 2.4. False-Positive Report Probability (FPRP) Analysis

The significant results were also assessed by the false-positive report probability (FPRP) [42]. We set 0.5 as FPRP threshold and assigned a prior probability of 0.1 to detect an odds ratio (OR) of 1.50 for an association with genotypes under investigation. Only the significant finding with a FPRP value < 0.5 was considered as a noteworthy result.

### 2.5. Statistical Analysis

STATA 11.0 (College Station, Texas 77845, United States) was used to calculate the pooled ORs along with corresponding 95% confidence intervals (95% CIs), in order to examine the correlation between RAD51 SNPs and the risk of these two cancers. The allelic, homozygous, heterozygous, dominant, and recessive genetic models were examined in our meta-analysis. Subgroup analyses based on genotyping method, ethnicity, sample size (large sample was defined by the overall number of cases and controls greater than 400; small sample was defined by the sample size equal to 400 or less than 400), and tumor type were conducted. The value of *P* < 0.05 in *Z*-test was regarded as statistically significant.

Heterogeneity was evaluated by Cochran's *Q*-statistic and *I*^2^ test. If *P* value in *Q*‐test < 0.10 or *I*^2^ > 50%, the DerSimonian and Laird random-effects model was used to count the ORs. Otherwise, the Mantel-Haenszel fixed-effects model was conducted to assess the correlation between RAD51 SNPs and these two cancers. Sensitivity analysis was performed by deleting an article at a time in order to evaluate the reliability and stability of the pooled results. Publication bias was evaluated by Begg's funnel plots and Egger's tests. If the *P* value < 0.05, the publication bias was considered significant.

## 3. Results

### 3.1. Eligible Papers and Paper Characteristics

**Figure 1:**
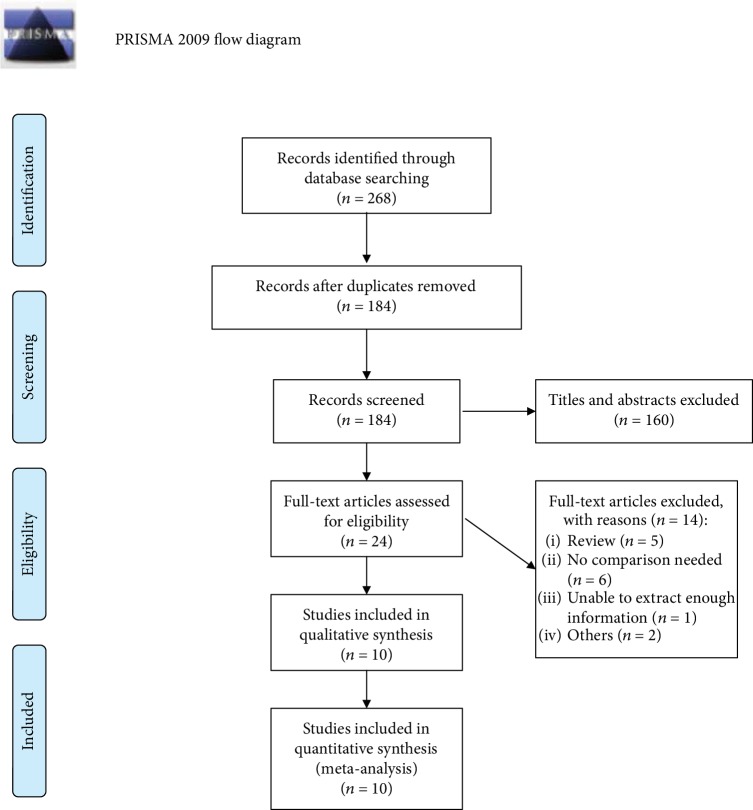
The progress of study selection.

According to the inclusion and exclusion criteria, ten papers involving a total of 2484 controls and 2377 cases were enrolled in our analysis after selection [29, 31–39]. The progress of paper selection is demonstrated in Figure 1.

All the enrolled papers were related to RAD51 G135C (rs1801320) polymorphism. Among ten articles, four were correlated with RAD51 G172T (rs1801321) polymorphism. There are seven articles for HNC and three articles for EC. Five articles were conducted in Caucasians; four articles were performed on Asians. All papers showed that the genotype distribution in the controls was consistent with HWE, except 2 papers [34, 39]. The detailed data of these enrolled articles are presented in Tables 1 and 2.

### 3.2. Results of Meta-Analysis

The correlation between the RAD51 polymorphisms and these two cancers is displayed in Tables 3 and 4.

**Figure 2:**
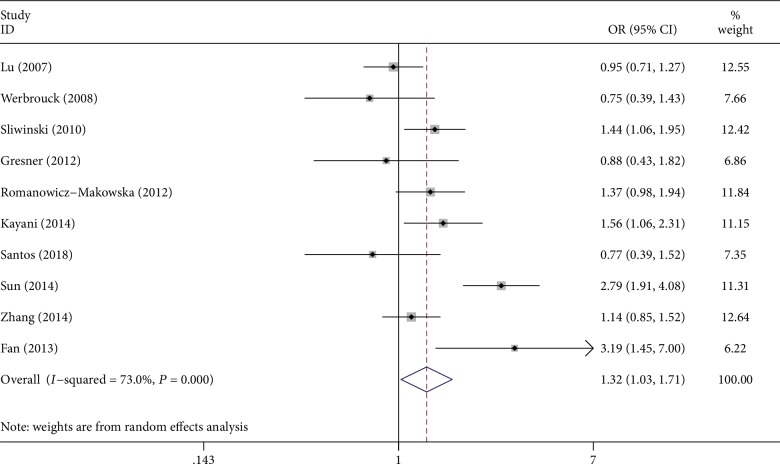
Forest plot for the association of rs1801320 polymorphism and two associated cancer risks under the allelic genetic model.

With respect to RAD51 G135C (rs1801320) polymorphism, the random-effects model was performed under all genetic models because the statistical heterogeneity between articles was substantial (value of *P* in *Q*‐test < 0.10 or *I*^2^ > 50%). We discovered a significant relationship between the rs1801320 polymorphism and the increased risk of these two cancers under all genetic models except homozygous and recessive models (OR = 1.32, (95%CI, *P*) = (1.03‐1.71, 0.032) for C vs. G, Figure 2; OR = 1.34, (95%CI, *P*) = (0.69‐2.61, 0.388) for CC vs. GG; OR = 1.50, (95%CI, *P*) = (1.03‐2.19, 0.033) for CG vs. GG; OR = 1.44, (95%CI, *P*) = (1.05‐1.99, 0.026) for CC+CG vs. GG; and OR = 1.15, (95%CI, *P*) = (0.57‐2.34, 0.696) for CC vs. CG+GG). In our subgroup analyses, a statistically significant correlation was observed for PCR-RFLP genotyping method subgroup (OR = 1.52, 95%CI = 1.15‐2.02 for C vs. G; OR = 1.84, 95%CI = 1.19‐2.86 for CG vs. GG; and OR = 1.74, 95%CI = 1.20‐2.51 for CC+CG vs. GG), Asians (OR = 1.89, 95%CI = 1.16‐3.08 for C vs. G; OR = 2.20, 95%CI = 1.30‐3.74 for CC vs. GG; OR = 2.06, 95%CI = 1.13‐3.76 for CG vs. GG; OR = 2.06, 95%CI = 1.17‐3.64 for CC+CG vs. GG; and OR = 1.89, 95%CI = 1.12‐3.19 for CC vs. CG+GG), large sample subgroup (OR = 1.41, 95%CI = 1.02‐1.96 for C vs. G; OR = 1.75, 95%CI = 1.01‐3.01 for CG vs. GG; and OR = 1.61, 95%CI = 1.04‐2.51 for CC+CG vs. GG), and EC (OR = 2.07, 95%CI = 1.01‐4.25 for C vs. G; OR = 2.08, 95%CI = 1.17‐3.71 for CC vs. GG; and OR = 1.78, 95%CI = 1.00‐3.15 for CC vs. CG+GG).

As for RAD51 G172T (rs1801321) polymorphism, four papers enrolled were all related to HNC instead of EC. The random-effects model was performed under all genetic models, because the statistical heterogeneity between articles was substantial (value of *P* in *Q*‐test < 0.10 or *I*^2^ > 50%). Significant correlation was not observed between rs1801321 polymorphism and susceptibility to HNC. In our subgroup analyses by genotyping method, ethnicity, and sample size, a significant correlation could be observed between the rs1801321 polymorphism and the decreased risk of HNC for Caucasians only (OR (95%CI) = 0.82 (0.72‐0.95) for T vs. G).

### 3.3. Sensitivity Analyses and Publication Bias

**Figure 3:**
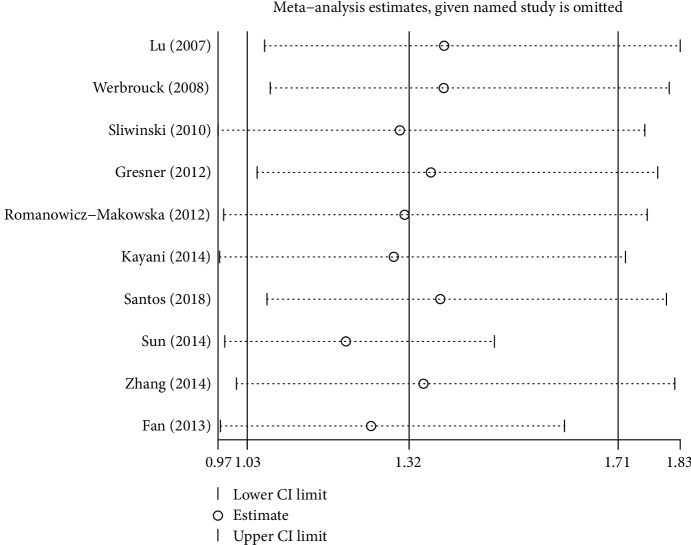
Sensitivity analysis of the pooled OR coefficients on the association for the rs1801320 polymorphism with two associated cancer risks under the allele model.

**Figure 4:**
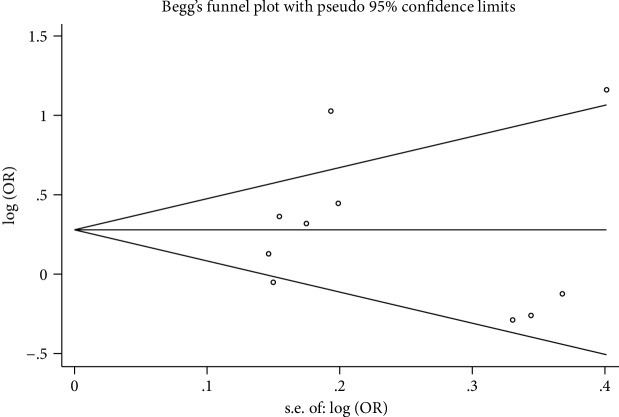
Funnel plot of publication bias for rs1801320 SNP with two associated cancers under the allele model.

Sensitivity analyses indicated that there was no substantive change in the combined ORs after excluding each article at a time (Figure 3, C vs. G of rs1801320). Publication bias was assessed by the Egger linear regression tests and Begg's funnel plots. In all studies, no remarkable publication bias was shown by the *P* value in the Egger test (C vs. G: *P* = 0.980; CC vs. GG: *P* = 0.299; CG vs. GG: *P* = 0.710; CC+CG vs. GG: *P* = 0.848; CC vs. CG+GG: *P* = 0.374; T vs. G: *P* = 0.540; TT vs. GG: *P* = 0.579; TG vs. GG: *P* = 0.669; TT+TG vs. GG: *P* = 0.625; TT vs. TG+GG: *P* = 0.595) and Begg's funnel plot (Figure 4, C vs. G of rs1801320) for rs1801320 and rs1801321 polymorphisms.

### 3.4. FPRP Analysis Results

The results of FPRP analyses for all discovered significant findings are listed in Table 5. For a prior probability of 0.1, the FPRP values were most less than 0.50 in the significant findings, suggesting that the most of these significant relationships were noteworthy although the FPRP values were more than 0.50 in three subgroup analysis (C versus G: in the EC subgroup; CG versus GG: in the large sample subgroup; CC versus CG+GG: in the EC subgroup).

## 4. Discussion

Reduced DNA repair capacity might lead to genomic instability and eventually result in tumorigenesis, which has aroused widespread concern. The RAD51 protein in human plays an essential role in repairing DNA breaks and maintaining the genetic steady. Two common RAD51 SNPs (rs1801320 and rs1801321) might influence mRNA stability and relate to the expression level of RAD51 protein.

In recent years, a number of papers have focused on the correlation between HNC, EC, and RAD51 gene polymorphisms. However, the observed correlations of these papers between two cancers and RAD51 SNPs were inconclusive. Therefore, we conducted a meta-analysis to assess the correlation between RAD51 SNPs and the risk of HNC and EC. Our results revealed that rs1801320 polymorphism was significantly correlated to the risk of these two cancers under the allelic, heterozygous, and dominant genetic models. Nevertheless, no association between the rs1801321 polymorphism and the development of HNC was found.

In the subgroup analyses of rs1801320 polymorphism according to a genotyping method, a statistically significant relationship was found in the PCR-RFLP genotyping method subgroup under allelic, heterozygous, and dominant genetic models, but not in the PCR genotyping method subgroup. This might be because different genotyping method would influence the association, indicating that a genotyping method with high sensitivity and specificity is required to increase the accuracy of results. In the subgroup analyses based on ethnicity, we discovered rs1801320 polymorphism increased the risk of HNC and EC in Asian populations, but not in Caucasian populations. This might be due to the following reasons. Firstly, it might be because the genetic trait was considerably different in various ethnicities. Secondly, it could be because the individuals' genetic susceptibility and environmental factors are diverse in different ethnic groups. When stratified by sample size, the rs1801320 polymorphism was related to the increased risk of HNC and EC in a large sample subgroup under the allelic, heterozygous, and dominant genetic models, which was the same as the overall results. Nevertheless, no marked association was discovered in a small sample subgroup. This might suggest that more studies with a large sample size are required to assess the correlation between RAD51 SNPs and the susceptibility to HNC and EC. Stratified analysis according to tumor type indicated that a marked correlation could be found between rs1801320 polymorphism and EC under the allelic, homozygous, and recessive genetic models, but not found between the SNP and the HNC. This indicated that rs1801320 polymorphism would contribute to the development of EC.

In the subgroup analyses of rs1801321 polymorphism by a genotyping method and sample size, no statistical evidence of correlation was observed between rs1801321 SNP and HNC. Stratified analysis based on ethnicity, our results revealed that rs1801321 SNP was related to the decreased risk of HNC among Caucasian populations under an allelic genetic model.

To the best of our knowledge, our study is proposed as the first one to observe the correlation between the RAD51 gene polymorphisms and the susceptibility to these two associated cancers. It is particularly worth mentioning here that rs1801320 polymorphism could increase the risk of EC, which had not been shown before. A previous meta-analysis carried out by Kong et al. [30] was performed to assess the effect of RAD51 polymorphisms on the susceptibility to HNC. In addition, some other meta-analyses were conducted to study the correlation between RAD51 polymorphisms and cancers including HNC [43–45]. Comparing with them, our analysis has some differences and improvements. Firstly, no correlations were observed between RAD51 gene polymorphisms and HNC, which did not correspond with previous studies [30, 43, 44]. Secondly, this is the first study not only to assess the correlation between RAD51 SNPs and two associated cancers (HNC and EC) but also to discover a relationship between the rs1801320 polymorphism and the risk of EC. Thirdly, we performed an updated study with more comprehensive data. Additionally, we conducted the FPRP analysis, and the results of FPRP analysis showed that most of the significant findings in our study are robust. There is no doubt that our present results will be more reliable.

However, some limitations exited in our analysis. First, the subgroup analyses could not have sufficient statistical power to identify the association because of the limited studies. Second, some eligible studies that have not published were not included in the present analysis, so publication bias could potentially exist. Third, subgroup analyses based on gender, smoking, or alcohol consumption were not performed since relevant data could not be obtained from most of the enrolled studies. Finally, the FPRP analysis results showed that the significant relationships of three subgroup analysis were not noteworthy (C versus G: in the EC subgroup, CG versus GG: in the large sample subgroup, and CC versus CG+GG: in the EC subgroup). So further analyses are required to investigate the effects on the gene-environment interaction.

## 5. Conclusions

The present meta-analysis explored that rs1801320 SNP was significantly correlated with the risk of these two associated cancers. Moreover, the correlation between the rs1801320 SNP and the susceptibility to EC was pointed out at the first time. However, more high-quality papers with a large sample size should be addressed to assess the correlation between RAD51 polymorphisms and cancer susceptibility.

## Figures and Tables

**Table 1:** The detailed characteristics of included studies.

First name (year)	Country	Ethnicity	Control type	Sample size cases/controls	Genotyping method	Cancer type
Lu (2007)	USA	Caucasian	HCC	716/719	PCR-RFLP	HNC
Werbrouck (2008)	Belgium	Caucasian	HCC	152/157	PCR	HNC
Sliwinski (2010)	Poland	Caucasian	HCC	191/353	PCR-RFLP	HNC
Gresner (2012)	Poland	Caucasian	PCC	81/111	PCR	HNC
Romanowicz-Makowska (2012)	Poland	Caucasian	PCC	253/253	PCR-RFLP	HNC
Xue-Jiao (2013)	China	Asian	HCC	123/61	PCR-RFLP	EC
Shu-Xiang (2014)	China	Asian	PCC	316/316	PCR-RFLP	EC
Kayani (2014)	Pakistan	Asian	HCC	200/150	PCR-RFLP	HNC
Ming-Zhong (2014)	China	Asian	HCC	219/258	PCR-RFLP	EC
Santos (2018)	Brazil	Unknown	HCC	126/130	TaqMan	HNC

HCC: hospital-based case control; PCC: population-based case control; PCR: polymerase chain reaction; RFLP: restriction fragment length polymorphism.

**Table 2:** Characteristics of included studies for RAD51 polymorphisms.

SNP	First name (year)	Cases (GG/GC/CC)	Controls (GG/GC/CC)	Cases (G/C-allele)	Controls (G/C-allele)	*P* (HWE)	NOS score
135G/C	Lu (2007)	624/91/1	622/96/1	1339/93	1340/98	0.170	7
	Werbrouck (2008)	136/15/1	134/23/0	287/17	291/23	0.322	7
	Sliwinski (2010)	101/88/2	258/64/32	290/92	580/128	<0.001	8
	Gresner (2012)	67/13/1	71/14/2	147/15	156/18	0.217	8
	Romanowicz-Makowska (2012)	174/69/10	190/58/5	417/89	438/68	0.816	7
	Xue-Jiao (2013)	83/35/5	54/6/1	201/45	114/8	0.123	6
	Shu-Xiang (2014)	206/100/10	216/92/8	512/120	524/108	0.626	7
	Kayani (2014)	120/70/10	106/41/3	310/90	253/47	0.674	7
	Ming-Zhong (2014)	144/56/19	223/24/11	344/94	470/46	<0.001	7
	Santos (2018)	110/16/0	111/17/2	236/16	239/21	0.174	8

		Cases (GG/GT/TT)	Controls (GG/GT/TT)	Cases (G/T-allele)	Controls (G/T-allele)		
172G/T	Lu (2007)	261/351/104	240/335/144	873/559	815/623	0.169	7
	Gresner (2012)	36/43/2	43/54/13	115/47	140/80	0.524	8
	Kayani (2014)	83/90/27	99/49/2	256/144	247/53	0.132	7
	Santos (2018)	51/52/23	51/56/23	154/98	158/102	0.271	8

**Table 3:** Results of overall and subgroup analyses for rs1801320.

	No	C versus G			CC versus GG			CG versus GG			CC+CG versus GG			CC versus CG+GG		
		OR	95% CI	P^(Z)^	OR	(95% CI)	P^(z)^	OR	(95% CI)	P^(z)^	OR	(95% CI)	P^(z)^	OR	(95% CI)	P^(z)^
Overall	10	1.32	1.03-1.71	0.032	1.34	0.69-2.61	0.388	1.50	1.03-2.19	0.033	1.44	1.05-1.99	0.026	1.15	0.57-2.34	0.696
PCR-RFLP	7	1.52	1.15-2.02	0.004	1.50	0.71-3.16	0.287	1.84	1.19-2.86	0.007	1.74	1.20-2.51	0.003	1.26	0.56-2.83	0.572
PCR	2	0.81	0.50-1.31	0.383	0.99	0.14-6.84	0.991	0.77	0.45-1.30	0.326	0.78	0.47-1.31	0.344	1.01	0.15-6.97	0.993
Caucasian	5	1.13	0.89-1.43	0.316	0.80	0.21-3.02	0.740	1.26	0.70-2.29	0.445	1.20	0.77-1.87	0.417	0.72	0.16-3.31	0.675
Asian	4	1.89	1.16-3.08	0.011	2.20	1.30-3.74	0.003	2.06	1.13-3.76	0.018	2.06	1.17-3.64	0.012	1.89	1.12-3.19	0.018
Large sample	5	1.41	1.02-1.96	0.040	1.18	0.45-3.06	0.741	1.75	1.01-3.01	0.045	1.61	1.04-2.51	0.033	0.98	0.35-2.80	0.976
Small sample	5	1.20	0.74-1.94	0.460	1.82	0.72-4.60	0.205	1.24	0.74-2.07	0.414	1.24	0.73-2.10	0.420	1.64	0.65-4.14	0.292
HNC	7	1.15	0.93-1.43	0.188	0.92	0.32-2.67	0.883	1.26	0.81-1.96	0.301	1.22	0.87-1.71	0.255	0.81	0.25-2.62	0.729
EC	3	2.07	1.01-4.25	0.048	2.08	1.17-3.71	0.013	2.38	0.96-5.89	0.060	2.31	1.00-5.35	0.050	1.78	1.00-3.15	0.049

**Table 4:** Results of overall and subgroup analyses for rs1801321.

	No	T versus G			TT versus GG			TG versus GG			TT+TG versus GG			TT versus TG+GG		
		OR	95% CI	P^(Z)^	OR	(95% CI)	P^(z)^	OR	(95% CI)	P^(z)^	OR	(95% CI)	P^(z)^	OR	(95% CI)	P^(z)^
Overall	4	1.11	0.66-1.87	0.686	1.12	0.37-3.40	0.846	1.17	0.78-1.76	0.451	1.16	0.67-2.03	0.592	1.05	0.40-2.76	0.926
PCR-RFLP	2	1.46	0.48-4.48	0.504	3.02	0.12-76.6	0.502	1.42	0.64-3.17	0.394	1.52	0.50-4.65	0.463	2.56	0.15-44.5	0.518
Caucasian	2	0.82	0.72-0.95	0.007	0.44	0.14-1.43	0.173	0.96	0.78-1.19	0.723	0.86	0.71-1.06	0.161	0.45	0.14-1.46	0.182
Small sample	3	1.24	0.57-2.66	0.590	1.43	0.17-12.2	0.743	1.27	0.71-2.30	0.424	1.30	0.59-2.86	0.520	1.31	0.19-8.87	0.782

**Table 5:** False-positive report probability values for the rs1801320 and rs1801321 gene polymorphisms.

Variables	OR (95% CI)	*P* ^a^	Power^b^	Prior probability				
				0.25	0.1	0.01	0.001	0.0001
rs1801320								
C versus G								
Overall	1.32 (1.03-1.71)	0.036	0.833	0.113	0.277	0.808	0.977	0.998
PCR-RFLP	1.52 (1.15-2.02)	0.004	0.464	0.025	0.070	0.455	0.894	0.988
Asian	1.89 (1.16-3.08)	0.011	0.177	0.153	0.351	0.856	0.984	0.998
Large sample	1.41 (1.02-1.96)	0.041	0.644	0.160	0.364	0.863	0.984	0.998
EC	2.07 (1.01-4.25)	0.047	0.190	0.428	0.692	0.961	0.996	1.000
CC versus GG								
Asian	2.20 (1.30-3.74)	0.004	0.079	0.120	0.291	0.819	0.979	0.998
EC	2.08 (1.17-3.71)	0.013	0.134	0.227	0.468	0.906	0.990	0.999
CG versus GG								
Overall	1.50 (1.03-2.19)	0.036	0.500	0.177	0.391	0.876	0.986	0.999
PCR-RFLP	1.84 (1.19-2.86)	0.007	0.182	0.100	0.250	0.786	0.974	0.997
Asian	2.20 (1.30-3.74)	0.004	0.079	0.120	0.291	0.819	0.979	0.998
Large sample	1.75 (1.01-3.01)	0.043	0.289	0.309	0.579	0.937	0.993	0.999
CC+CG versus GG								
Overall	1.44 (1.05-1.99)	0.027	0.598	0.120	0.290	0.818	0.978	0.998
PCR-RFLP	1.74 (1.20-2.51)	0.003	0.214	0.041	0.114	0.585	0.934	0.993
Asian	2.06 (1.17-3.64)	0.013	0.137	0.219	0.457	0.902	0.989	0.999
Large sample	1.61 (1.04-2.51)	0.036	0.377	0.220	0.459	0.903	0.989	0.999
CC versus CG+GG								
Asian	1.89 (1.12-3.19)	0.017	0.193	0.210	0.444	0.898	0.989	0.999
EC	1.78 (1.00-3.15)	0.048	0.278	0.340	0.607	0.944	0.994	0.999
rs1801321								
T versus G								
Caucasian	0.82 (0.72-0.95)	0.008	0.997	0.024	0.069	0.449	0.892	0.988

^a^Chi-square test was adopted to calculate the genotype frequency distributions. ^b^Statistical power was calculated using the number of observations in the subgroup and the OR and *P* values in this table.
